# Implementation of a health care policy: An analysis of barriers and facilitators to practice change

**DOI:** 10.1186/1472-6963-5-53

**Published:** 2005-08-15

**Authors:** Susan Watt, Wendy Sword, Paul Krueger

**Affiliations:** 1 School of Social Work, McMaster University, Hamilton, Ontario, Canada; 2 School of Nursing, McMaster University, Hamilton, Ontario, Canada; 3 Department of Clinical Epidemiology & Biostatistics, McMaster University, Hamilton and Senior Research Associate, St. Joseph's Health System Research Network, Brantford, Ontario, Canada

## Abstract

**Background:**

Governments often create policies that rely on implementation by arms length organizations and require practice changes on the part of different segments of the health care system without understanding the differences in and complexities of these agencies. In 2000, in response to publicity about the shortening length of postpartum hospital stay, the Ontario government created a universal program offering up to a 60-hour postpartum stay and a public health follow-up to mothers and newborn infants. The purpose of this paper is to examine how a health policy initiative was implemented in two different parts of a health care system and to analyze the barriers and facilitators to achieving practice change.

**Methods:**

The data reported came from two studies of postpartum health and service use in Ontario Canada. Data were collected from newly delivered mothers who had uncomplicated vaginal deliveries. The study samples were drawn from the same five purposefully selected hospitals for both studies. Questionnaires prior to discharge and structured telephone interviews at 4-weeks post discharge were used to collect data before and after policy implementation. Qualitative data were collected using focus groups with hospital and community-based health care practitioners and administrators at each site.

**Results:**

In both studies, the respondents reflected a population of women who experienced an "average" or non-eventful hospital-based, singleton vaginal delivery. The findings of the second study demonstrated wide variance in implementation of the offer of a 60-hour stay among the sites and focus groups revealed that none of the hospitals acknowledged the 60-hour stay as an official policy. The uptake of the offer of a 60-hour stay was unrelated to the rate of offer. The percentage of women with a hospital stay of less than 25 hours and the number with the guideline that the call be within 48 hours of hospital discharge. Public health telephone contact was high although variable in relation to compliance the guideline that the call be within 48 hours of hospital discharge. Home visits were offered at consistently high rates.

**Conclusion:**

Policy enactment is sometimes inadequate to stimulate practice changes in health care. Policy as a tool for practice change must thoughtfully address the organizational, professional, and social contexts within which the policy is to be implemented. These contexts can either facilitate or block implementation. Our examination of Ontario's universal postpartum program provides an example of differential implementation of a common policy intended to change post-natal care practices that reflects the differential influence of context on implementation.

## Background

A social policy expresses "ongoing strategies for structuring relationships and coordinating behaviour to achieve collective purposes...ways of exerting power, of getting people to do things that they might otherwise not do" [1]. The implementation of a policy requires that resources come from wherever necessary to enact the relevant program(s) and "that the economic structure, social institutions, and political processes will be shaped to protect and maintain that commitment" [2]. The purpose of this paper is to examine how a health policy initiative was implemented in two different parts of a single payer, government run health care system and to analyze the barriers and facilitators to achieving practice change.

The implementation of the 1999 policy-expansion of the Healthy Babies Healthy Children (HBHC) program, under the aegis of the government in Ontario, Canada, provided an instructive example of a policy intended to drive health care practice. The expansion of this universal program extended to women the option of staying in hospital for 60 hours following an uncomplicated vaginal birth for "assessment, support and follow-up purposes" [3]. The stated goal of the policy was to give "women some flexibility" in length of stay (LOS) in hospital after childbirth and to provide enhanced community-based postpartum services. This policy was in keeping with the 1996 recommendations of The Canadian Pediatric Society and the Society of Obstetricians and Gynecologists of Canada [4].

The policy prescribed a province-wide implementation mechanism for the community care portion of the policy that consisted of a public health initiated telephone call within 48 hours of postpartum discharge and the offer of a home visit to all mothers, regardless of their LOS, demographic profile or residential location in the province [3]. No specific implementation mechanism was prescribed for extending the LOS; hospitals were charged with developing their own implementation plans.

Grol and Grimshaw [5] categorized some of the environmental factors that may become barriers to the implementation of evidence in practice. The universal extension of the HBHC program through two distinctive components of the health care system, with different structural and functional accountabilities, provides an interesting opportunity to examine how contextual factors can act as barriers or facilitators, leading to very different implementation strategies and uptake levels.

These groupings provide a reasonable way of examining barriers to policy implementation at the practice level. The organizational, professional, and social contexts into which a policy is introduced may block or facilitate practice change. What happens at the level of implementation when a government imposes universal changes in practice standards not driven by, or even agreed to by, clinicians? What happens when the implementation of a policy is dependent upon two quite different segments of the health care system – hospitals and public health units – each sector controlled by different professions and operating from related, but different, mandates? What happens when women who are the objects of the policy are neither consulted nor required to be informed in any systematic way about the policy?

Far from being a new phenomenon, postpartum "early release" from hospitals for healthy mothers and newborn infants has been a contentious but familiar theme in both the practice and the politics of Canadian health care in the twentieth century [6]. By the mid twentieth century, in-hospital birth and postpartum care became the norm in North America [6]. This change in location from community to hospital was driven by a concern for the mother's health as much as a concern about infant mortality or morbidity. [7]

The shift from home deliveries to a hospital setting reached a peak in the 1960s [8,9]. Within the first 50 years of the twentieth century, the high and well-known numbers of maternal deaths and injuries pushed physicians to do what they did best – to medicalize pregnancy and childbirth [6]. As with most medical advice, this practice was couched in terms of safety for mothers and infants; [8,10]. Childbirth became firmly lodged within the mandate and practices of the 'sickness care system' [11].

Physicians prescribed 'lying in' for 14 days postpartum, in spite of evidence that suggested shorter hospital stays and getting out of bed sooner were better for the health of women [6,8]. However, by 1949, according to Mitchinson, postpartum stays had decreased to an average of 10 days post delivery. 'Early rising' began to be debated and occasionally practiced during this time, mostly popularized by the post WWII increase in births and concurrent shortage of hospital beds [6].

From the 1970s through the 1990s, postpartum hospital stays shortened. In Canada, Wen reported the mean length of hospital stay after delivery decreased from 5.3 days in 1984–85 to 3.0 days in 1994–95[12]. In response to an expressed wish on the part of women and their partners, the practice of family-centred obstetrics led hospitals to develop early discharge programs to accommodate strong patient desires [8]. By 1999, the average length of stay for a "delivery in a completely normal case" (Code 650) was 1.8 days [13]. These developments mirrored similar changes in practice in the U.S. [14]. Decter reports that, concurrent to this, hospital stays for a variety of procedures and illnesses were becoming shorter as well due in part to emerging technology and changing practice standards and in part to fiscal restraints [15].

Similarly, public health nursing practice changed over time. In the 20^th ^century, the mandate of public health expanded to specifically include improving maternal child health [16]. Public health continued to place emphasis on the health and well being of mothers and infants in many ways (e.g., 1998 Ontario Healthy Babies, Healthy Children Program). This program is a prevention and early intervention initiative intended to provide support and services to families with children from before birth up to six years of age that could benefit from additional resources. At the time the universal postpartum program was introduced, the HBHC program was already in place, with public health nurses engaged in calling and providing home visits for mothers and infants identified through in-hospital screening [17].

The 1990s were troubled years for the Canadian healthcare system and, consequently, for the governments which managed provincial operations [18,19]. Increasingly, hospitals cared only for critically ill patients; convalescence occurred in the community with or without the support of community-based health care providers. At the same time, public health units in Ontario stopped routine visits to newborn infants and their mothers. By early 1998, an Ipsos-Reid poll found that 73% of Canadians believed that the healthcare system was worse than it had been 5 years previously [20]. Consumers and potential consumers of medical care services were expressing the fear that reductions in in-hospital care were compromising patient well-being.

These concerns extended to postpartum and newborn care. In Canada, in 1997, there occurred what has been defined as a "focussing event" – something that draws widespread attention and publicity to an existing problem [21,22]. The accidental death of a newborn infant from dehydration led to a Coroner's inquest that raised questions about a connection between the death of a seemingly healthy infant and maternity short stay hospital policies [23].

The political lessons from this event were not lost on a Canadian government with a health care system under criticism. By 1998, the Government of Canada had created Family-Centred Maternity and Newborn Care: National Guidelines [24]. The guidelines made clear that, while early discharge programs had been judged safe, satisfying, efficient and economical for their users, the success of these programs rested on the following factors: parental choice, post-natal screening, community support components, and appropriately trained professional staff [24]. They go on to say that where "administrative mandates may give rise to a non-voluntary, short hospital stay", this must be coupled with community support strategies and a program of community follow-up care [[24] 6.36]. It is very clearly stated that "the mother...should decide the length of hospital stay, based on her individual needs" [[24] 6.36].

Facing an election call the Conservative Government of Ontario announced in their 1999–2000 business plan [25] their intention to give additional funding to hospitals for extending the stay of postpartum women and to the HBHC program for a postpartum telephone call and home visit [3]. This universal policy had the advantage of being potentially popular, apparently caring, medically harmless, relatively inexpensive and appealing to an already concerned public, media and professional community, even though evidence of efficacy or system capacity to implement the policy was not clear.

The Ontario Mother and Infant Survey (TOMIS) of mother and infant health and service utilization was completed just prior to the introduction of this policy. A replication study, TOMIS II, provided an opportunity to examine the outcomes of this policy and to search for the factors that shaped the uptake of the policy by providers and consumers.

## Methods

The context of our examination of the implementation of this policy was two research studies, one initiated before and one after the HBHC policy enhancement. The primary methodology used in both studies was a cross-sectional survey.

Data collection for TOMIS occurred between November 1998 and June 1999 just prior to the Hospital Stay and Postpartum Home Visiting Program extension to the HBHC program in November 1999. This provided us with baseline data to compare with findings from TOMIS II. These data were collected from September 2001 to June 2002.

The survey methods and instruments used for TOMIS II paralleled those used in TOMIS, which allowed for an appropriate comparison of data at two points in time. The same study sites, sample size, eligibility criteria, recruitment strategy, and instruments were used for the two surveys[13,14]. In both studies, women completed a questionnaire before discharge from hospital and participated in a structured telephone survey at 4-weeks post-discharge.

Five purposefully selected Ontario hospitals provided respondents who constituted a cross-section of mothers and newborn infants with diverse socio-economic characteristics and access to varying health and social services. The characteristics of the hospitals are presented in Table 5

**Table 5:** 

Site 1	Southern, suburban, teaching hospital, metropolitan catchment area, 3900 annual births
Site 2	Central east regional centre, urban & rural catchment areas, 1500 annual births
Site 3	Central south regional centre, urban & rural catchment areas, 4500 annual births
Site 4	Southern, urban, teaching, metropolitan catchment area, 2700 annual births
Site 5	Central north regional centre, urban & rural catchment areas, 2000 annual births.

Participants for both studies included the first 250 eligible, consenting women from each site, totaling 1,250 participants in each study. This sample size was determined to be large enough to allow for the examination of many variables together, and was in keeping with the generally accepted guideline of 30 subjects per variable[15]. Women were eligible if they (a) had given birth vaginally to a single live infant, (b) were being discharged from hospital at the same time as their infant, (c) were assuming care of their infant at the time of discharge, and (d) were competent to give consent to participate. Women were excluded if they (a) had an infant who required admission to a neonatal intensive care or special care nursery for more than 24 hours or (b) were unable to communicate in one of the study languages – English, French, Chinese, and Spanish. Each study hospital continued to utilize its own postpartum care protocols throughout the recruitment period. Participants received services from the public health units related to their residence, which by and large, were those located in the same geographic region as the hospital sites. A full description of the methodology has been previously published[14]. The ethics review committees of McMaster University and each of the hospitals involved in the study granted ethical approval.

Descriptive statistics were computed by site for all variables measured, including frequency counts and percentages. Chi-square tests were used to determine differences between sites or differences between TOMIS and TOMIS II data. A probability level of <0.05 was used to determine statistical significance. SPSS was used for all statistical computations.

In both of the studies, following preliminary analysis of the survey findings, focus groups were held at each of the sites. These groups were comprised of front-line clinical and administrative staff from each hospital and community agencies. TOMIS II focus group participants were asked to reflect not only on the findings in the context of local practices and policies, but also on local implementation of the universal Hospital Stay and Postpartum Home Visiting Program. They were asked to comment specifically on the extent to which the program had been implemented in their community and implementation challenges. It therefore is only the TOMIS II focus group findings that are relevant to this paper.

One TOMIS II site was unable to participate in the focus groups. At three of the four sites that did participate, we were able to hold a focus group for only community-based providers and managers, and a second focus group for only hospital-based participants. Due to planning issues, the two focus groups at the fourth site were a combination of community and hospital personnel. The size of the focus groups ranged from 8 to 12 individuals. Each group interview was about an hour long, and was audio taped and later transcribed verbatim.

In TOMIS II, focus group participants, in addition to commenting on the survey findings, were asked to describe local implementation of the universal Hospital Stay and Postpartum Home Visiting Program. They were asked to comment specifically on the extent to which the program had been implemented in their institution or community and on the implementation challenges that they had experienced.

Focus group data were analyzed using an inductive approach. Two research assistants independently coded the transcripts, with phrases and sentences that described specific aspects of program implementation being given a descriptive code. The research assistants then met and reached consensus on a coding scheme that resulted in the assignment of a common code to data that were similar. The emergent themes were reviewed and validated with one of the research team members.

## Results

### Information providers

In both TOMIS and TOMIS II there were no statistically significant differences in the sociodemographic characteristics of those recruited in hospital and those who completed the telephone interviews 4-weeks post discharge [26]. No anomalous results in relation to these characteristics were found when comparisons were made to the statistical profiles developed from Statistics Canada data about women ages 15 to 45 in each of the communities served by each hospital. Infants born to study participants were full-term and of normal birth weight [26]. Further, the focus groups endorsed the representativeness of the sample of the population of clients at each site. Therefore, the authors are confident that the respondent groups in both studies reflect a population of women who experienced an "average" or non-eventful hospital-based, vaginal, singleton delivery.

Focus groups were held at each site except Site 4. Participants in TOMIS II focus groups included nursing and physician administrators from obstetrical units in the site hospital, front-line nurses, lactation consultants, clinical educators, social workers, midwives, and public health nurses and nursing administrators. A total of approximately 80 people participated in these focus groups.

### Length of stay

In TOMIS II, the findings shown in Table 1 demonstrate wide variance in the implementation of the offer of 60-hour stay among the sites. It is possible that characteristics of mothers and/or newborn infants could explain this variance. Although the offer was associated with a younger maternal age, first live birth, self-identified ethnicity as Canadian, English or French as 1^st ^language, and maternal place of birth as Canada, these sociodemographic characteristics failed to explain the site variability [33]. Explanation was sought, therefore, in the sites' implementation of the policy.

**Table 1:** Offer and Acceptance of 60-hour Length of Stay

	**Site 1 ** No. (%)	**Site 2 ** No. (%)	**Site 3 ** No. (%)	**Site 4 ** No. (%)	**Site 5 ** No. (%)
Offered a 60-hr stay ^a b^	20 (11.7)	78 (41.9)	168 (81.2)	69 (39.9)	80 (52.3)
Accepted a 60-hr stay^c^	4 (21.1)	28 (39.4)	51 (30.4)	21 (31.3)	17 (21.3)

^a ^Chi-square test indicated a statistically significant difference (P < 0.001) across sites for offer of a 60-hr stay

^b ^Offer is reported for those who took part in the scheduled telephone interview at 4 weeks post-discharge (n = 890)

^c ^Acceptance is reported for those offered a 60-hr stay (n = 405)

It became quite clear in the focus groups that hospital participants did not view the policy of offering a 60-hour stay as prescriptive. They talked about the policy as difficult to implement given the lack of beds in their hospitals and some were convinced that, given an option, women would choose to stay for longer than medically necessary.

Representatives of the hospital frequently expressed surprise that mothers even knew about the policy and at the rates at which women reported being offered a 60-hour stay. They disavowed any responsibility on the part of the hospital for informing women about the LOS aspect of the policy. Some focus group participants were sure that, rather than being offered the 60-hour stay, women were "demanding 60 hours" because "we don't volunteer that information". Interestingly, focus group members sometimes attributed acknowledgement of an offer by a mother as "recall error" because "our policy is 2 days and out".

Focus group participants from the two sites with significantly higher offer rates (Sites 3 and 5) had a slightly different perspective when interpreting the results from their hospitals in comparison with other sites. They stated that they knew about the policy and while not necessarily agreeing with it, believed that they had an obligation to their patients to adhere to the policy. In neither site was information about the policy provided to women in a standard format; it was not part of the nursing admission protocol or available in writing to mothers. However, prenatal class teachers in both geographic areas were reported to have been telling expectant women in a consistent fashion that they were entitled to up to a 60-hour stay. Family physicians also were reported as a source of policy information for women. In both of these sites, nurses believed that women whom they viewed as being "at high risk" (e.g., teenage mothers, 1^st ^time breastfeeding mothers, mothers living at a significant distance from the hospital) received information about the 60-hour option from postpartum hospital staff but were unable to explain the basis of their belief in any formal information provision. One representative who said that women "heard about it somehow – if not from us directly then from other women in the unit" expressed the view of focus group participants from several sites.

The uptake of the offer of up to a 60-hour stay was between 21 and 39% (Table 1) and was unrelated to the rate of offer. Sociodemographic characteristics did not differentiate between those who took up the offer and those who did not. However, uptake was associated with first live birth, infant health problems, maternal health problems, and the mother having two or more concerns related to herself or her infant (See Sword, Watt, Krueger, 2004, [26] for more details). In short, mothers who felt less sure of their own ability to care for themselves and their newborn infant were more likely to stay longer in hospital. It is interesting to note that one of these sites had decided, following the completion of data collection, to no longer universally offer a 60-hour stay.

If the intent of this policy was to produce fewer "drive through" deliveries, then the appropriate question may not be "Were you offered/did you accept a 60-hour stay?" Perhaps the more important issue is, did the policy result in increased LOS overall, and particularly in a decrease in stay of less than 25 and 48 hours?

Table 2 shows that the percentage of women with a stay of less than 25 hours declined in all sites following policy implementation. Similarly, it demonstrates that there were fewer women with a stay of 48 hours or less. The result is that there was a shift to marginally longer lengths of stays for the two groups staying in hospital for the shortest periods.

**Table 2:** Length of Stay ^a^

	**LOS <25 hours**				**LOS ≤48 hours**				**LOS >48 hours**			
	
**Site**	**T1 %**	**T2 %**	**Change %**	**p-value**	**T1 %**	**T2 %**	**Change %**	**p-value**	**T1 %**	**T2 %**	**Change %**	**p-value**
**Site 1**	59.1	42.7	-16.4	0.005	98.7	91.8	-6.9	0.011	0.0	6.4	6.4	0.005
**Site 2**	11.0	9.7	-1.3	0.81	78.5	67.8	-10.7	0.028	15.5	15.1	-0.4	0.97
**Site 3**	32.5	12.6	-19.9	<0.001	91.8	62.8	-9.0	<0.001	4.3	23.2	18.9	<0.001
**Site 4**	45.3	25.9	-19.4	<0.001	94.2	80.4	-13.9	0.001	0.7	9.8	9.1	0.002
**Site 5**	23.6	13.1	-10.5	0.031	64.2	62.0	-2.2	0.79	14.5	10.5	-4.0	0.40

^a ^Chi-square tests were used to determine whether statistically significant differences existed between TI and T2

It appears that a shift in LOS occurred following the policy initiation. This change marginally increased the most common LOS to the 25 to 48 hour period by decreasing the incidence of discharge in less than 25 hours. Practice changed to become more in line with professional recommendations.

Regardless of whether or not they were offered or accepted up to a 60-hour stay, and unrelated to their actual LOS, most women stated that at the time of discharge they were ready to leave hospital. Similarly, 4 weeks after discharge they continued to see their stay as appropriate for their needs (Table 3 . Despite different approaches to deciding on an appropriate LOS at each site, site was not statistically associated with maternal readiness for discharge or satisfaction with their LOS. On the other hand, having a choice about LOS was associated with maternal satisfaction (p < 0.01).

### Public health initiated contact

The second aspect of the expansion of the HBHC program provided for contact with public health initially through a telephone call within 48 hours of discharge from hospital. During this phone call, a home visit to the mother and newborn infant by a public health nurse was to be offered. Women in TOMIS II reported very high rates of public health telephone contact (Table 4). There were significant drops in those rates when asked if the phone call had come within 48 hours of discharge.

**Table 4:** Public Health Initiated Contact ^a b^

	**Site 1 ** No. (%)	**Site 2 ** No. (%)	**Site 3 ** No. (%)	**Site 4 ** No. (%)	**Site 5 ** No. (%)	**p-value**
**Telephone call anytime after discharge**	150 (88.8)	178 (97.8)	180 (87.8)	136 (81.4)	143 (94.7)	p < 0.001
**Telephone call within 48 hrs of discharge**	125 (74.0)	135 (75.0)	131 (64.2)	119 (71.7)	120 (80.0)	p = 0.017
**Home visit offered**	143 (95.3)	161 (91.5)	169 (96.6)	129 (95.6)	135 (94.4)	p = 0.276
**Home visit accepted **^c^	109 (76.2)	72 (44.7)	69 (40.8)	93 (72.1)	89 (65.9)	p < 0.001

^a ^Chi-square tests were used to determine whether statistically significant differences existed between sites.

^b ^N = 890

^c ^Acceptance is reported for those offered a home visit

Focus group participants explained that lack of staff on weekends and problems with the transfer of information from hospitals to health units accounted for delays in phoning women. Home visits were offered to virtually all mothers but were accepted at highly variable rates ranging from 40.8% to 76.2% [26]. Focus group participants unanimously endorsed the policy initiative seeing it as a positive move for both their organizations and for women and infants. They acknowledged receipt of additional resources to implement the policy and were clear about the factors that inhibited complete compliance with the published service standards. Most often participants stated that a lack of resources was the primary reason accounting for low visitation rates. (For further discussion, see Sword, Watt, Krueger, 2004 [26].)

## Discussion

The policy addressed in this paper was intended by the government to be universally implemented. Its two parts – the offer of an option for mothers and infants to remain in hospital for up to 60 hours and public health contact through a telephone call within 48 hours of discharge and the offer of a home visit – were implemented at strikingly different rates in different locations by two sectors of a publicly funded, universal health care system.

In the hospital sector, the major barrier which appears to have influenced implementation is that of organizational context. Providers at two of the four hospital sites did not view extension of maternal LOS as a requirement of service delivery. Health care providers and administrators from these sites told us that there simply were not enough beds to allow for longer stays. In fact, since no extra operational funding came specifically to hospitals for this initiative, there was a financial disincentive to keep mothers longer. The dissenting sites reluctantly viewed the policy as an organizational requirement and consequently have high offer rates.

The downsizing of hospitals in general, and maternity wards in particular, were seen by focus group participants as having created a scarcity of beds to accommodate any increased LOS. They had anticipated high rates of acceptance of the offer and were convinced that resources were not available to implement the policy. Despite this reluctance hospitals lengthened their shortest stays suggesting that other factors were in play that modified organizational outcomes.

The general lengthening of stays is an important result because there is no evidence suggesting a longer LOS is universally beneficial. Although some studies have found a relationship between a shorter LOS and newborn infant readmission to hospital [27-29], others have not supported this relationship [9,30,31]. In sum, professional juries are still out on the optimal postpartum LOS for women who have uncomplicated deliveries.

The professional context provides some explanation for the implementation patterns. Some providers did not see themselves as responsible for informing women of the 60-hour stay option and, in fact, were surprised that women had knowledge of the policy and would exercise this option. They reflected a view of LOS as a clinical decision and one that is most appropriately made by mothers and practitioners rather than by policy makers. Providers were quick to point out that if they assessed a mother as being in need of more time in hospital, then she stayed longer. They saw part of their role as one of professional advocacy for patient services when they believed it necessary. Applying the policy would have meant giving up this discretionary professional activity. The association of longer stay with women who fall into traditional "high risk categories" suggests that discretionary offers were still being made and that the professional context modified organizational context in terms of implementation.

The dissonance created in hospital-based health care providers by this policy was evident when they talked about implementing the LOS policy. Their behaviours, including deliberately not telling women about the policy and actively discouraging extended stays, suggests the presence of barriers to implementation based on accepted standards of practice. For example, physicians remained in control of discharge orders and were reported by nursing focus group participants as unwilling to change their traditional practices. Lack of physician support for increasing LOS also accounted for the inability of nursing staff to see this policy as viable in their hospitals. Implementation of a change in-hospital health care policy relies on physician cooperation; they remain the final arbiters of most practice policy, including LOS.

What seemed to be at issue was who should make the decision and on what basis. Great concern was expressed by hospital providers that if women were given the option of staying longer in hospital, most would do so. That impression was not upheld by the responses of women in the study. Generally, women were eager to be discharged from hospital when they felt well and when they perceived their infants to be well. Even when offered an extended stay, most did not accept and whether or not they were offered a longer stay, they found their LOS to have met their needs.

Women's approaches to determining how long to stay in hospital provide one measure of the social context, consumer expectations. In this instance, perceptions of patient knowledge about the policy and expectation regarding LOS was influential in supporting implementation even in the face of organizational and professional contexts which erected strong implementation barriers.

These findings suggest that if implementation is to be successful, all the players need to be included. Consumers need to know about health policies and, if not involved in the formulation of the policy, at least systematically informed of their health care options. The provision of choice and active decision making appears to promote consumer satisfaction, at least in the instance of satisfaction with postpartum LOS.

On the other hand, the section of the policy extension that affected public health practice was implemented consistently and at a high rate at all sites. The strengthening of the community follow-up for mothers and newborn infants by public health nurses had a clearly identifiable proponent and implementation strategy. Direct funding flowed to health units specifically targeted for the provision of these additional services. Public health units received additional resources to provide a service that they had long wanted to offer in their communities, were trained to provide, and believed in professionally.

According to the Ministry responsible, this service expansion did not come at a cost to other public health services although some focus group participants did not share this view. We can only speculate about what might have happened if more women had accepted the offer of a home visit thereby placing more demands on the finite resources of public health units. However, the policy did not establish a specific goal of doing home visits, but rather of offering home visits. In this regard, public health units met the offer-target at all of the sites.

Direct accountability for implementation was assigned by the policy to public health care professionals who believed in the initiative and had been advocates for its adoption. In practice, the lack of weekend staff which public health units claimed occurred because of inadequate funding compromised the ability of providers to attain the specified timeframe for telephone contact, but did not lower the overall rate of contact or appear to interfere with the offer of home visits. Unlike hospitals that had been working with a goal of reducing LOS and fewer resources, health units found the policy to be consonant with their overall mission and feasible with the additional provincially provided resources. Also, unlike extending LOS, the approval of individual physician providers was not needed to implement this aspect of the policy. In addition to adequate funding, only the support of public health practitioners, who already advocated for this approach, was required for implementation. In short, the policy was consonant with the organizational, professional, and social contexts of public health practice and came with adequate resources to meet professionally endorsed implementation targets with strong historic roots.

In this instance women as service consumers again responded variably. Uptake of the offer of a home visit appear to have reflected women's perceptions of their own needs and a home visit by public health nurses as an appropriate way to meet those needs with a range of variables at play in determining these perceptions [26].

## Conclusion

Policy implementation in any health care system relies upon provider commitment. Policies that do not address the organizational, professional and social contexts are unlikely to achieve successful implementation. Political objectives alone, however well intentioned, are inadequate to change practice. When barriers to policy implementation exist in any of these contexts, the policy may fail to meet its objectives.

The common goal of positive health outcomes is shared by providers, consumers, and policy makers. Policy makers in any system must respect the knowledge and experience of providers when developing policies that require practice change. Providers need to appreciate and endorse changes in practice, to be "on board" with at least the intent of the policy; they need to value, support, and act on any policy entitlement. Consumers need to be informed and prepared to hold both providers and policy makers accountable in the making and implementing of health policy. A consumer right, in the absence of provider responsibility and accountability, appears to lead to the implementation of only those aspects of a health policy with which providers agree and for which there are perceived to be adequate resources.

Often in health care, providers are cast in the role of service gatekeepers. When policies increase consumer entitlement, they also challenge the authority of the gatekeeper role. Therefore, any universal policy that challenges this role needs both policy makers and practitioners to view it not as a suggestion but rather as a requirement. There must be consequences for failure to comply with the policy. At the same time, providers must be convinced that the policy can be implemented and that the outcome will be positive. If implementation is to be successful, policy makers need to engage providers in the process of policy development by acknowledging and entering into the contexts of the providers.

In the case of the LOS policy examined in this paper, providers were not obligated to action. The policy was merely a statement in principle, leaving action largely on the shoulders of postpartum women themselves. The policy was permissive rather than prescriptive. It relied on hospitals and healthcare providers to act often against the pressures of organizational, professional, and social contexts such as shorter stays, prevailing practice trends, and patient expectations. In such circumstances it seems unlikely that the policy will be implemented.

It would appear that policy statements, no matter how convincing, cannot be assumed to change health care practice. Other facilitating and inhibiting factors must be addressed if policy is to be used as a tool to change practice. Policy makers need to carefully consider not only the intent and objectives of a policy, and the evidence for and against alternative approaches, but also the contextual barriers faced by policy implementers.

## Competing interests

The author(s) declare that they have no competing interests.

## Authors' contributions

SW had a major role in designing the study and writing the proposal, co-supervised all aspects of study implementation, participated in data analysis, and was the lead writer of this manuscript.

WS had a major role in designing the study and writing the proposal, co-supervised all aspects of study implementation, participated in data analysis, contributed to the manuscript, and provided editorial comments.

PK contributed to the study design, implementation, analysis and interpretation, as well as the writing of the manuscript.

**Table 3:** Mother's Readiness for Discharge

	**In hospital**		**4 weeks post discharge**	
	
	**N (1237)**	**%**	**N (888)**	**%**
**Yes (Definitely & probably)**	1055	85.3	786	88.5
**No (Not sure, Definitely & probably)**	182	14.7	102	11.5

## Pre-publication history

The pre-publication history for this paper can be accessed here:


